# Bacterial Communities in Lanna Phak-Gard-Dong (Pickled Mustard Green) from Three Different Ethnolinguistic Groups in Northern Thailand

**DOI:** 10.3390/biology11010150

**Published:** 2022-01-17

**Authors:** Rujipas Yongsawas, Angkana Inta, Jatupol Kampuansai, Hataichanok Pandith, Nakarin Suwannarach, Saisamorn Lamyong, Panuwan Chantawannakul, Thararat Chitov, Terd Disayathanoowat

**Affiliations:** 1 Department of Biology, Faculty of Science, Chiang Mai University, Chiang Mai 50200, Thailand; rujipas_y@cmu.ac.th (R.Y.); angkhana.inta@cmu.ac.th (A.I.); Jatupol.K@cmu.ac.th (J.K.); hataichanok.p@cmu.ac.th (H.P.); suwan.462@gmail.com (N.S.); saisamorn.l@cmu.ac.th (S.L.); panuwan.c@cmu.ac.th (P.C.); thararat.chitov@cmu.ac.th (T.C.); 2 Research Center in Bioresources for Agriculture, Industry and Medicine, Chiang Mai University, Chiang Mai 50200, Thailand; 3 Research Center of Microbial Diversity and Sustainable Utilization, Chiang Mai University, Chiang Mai 50200, Thailand

**Keywords:** *Brassica juncea*, ethnic, fermented food, metagenomic data, microbiome, pickled mustard green, pickles

## Abstract

**Simple Summary:**

Phak-gard-dong (PGD), or pickled mustard green, is one of the most popular ethnic foods in Northern Thailand. Many bacteria are involved in its fermentation process. In this study, we explore the bacterial communities in PGD of three different ethnolinguistic groups of northern Thailand, including Karen, Lawa, and Shan. Moreover, we evaluate the potential of PGD as a functional food. The result of this study demonstrated two major bacteria that have been reported to be beneficial to human health. Bacterial compositions in PGD of the Lawa were distinctive from the other ethnic groups. This warrants the necessity of conservation of PGD and the development of this indigenous fermented food into a variety of health food products.

**Abstract:**

The Lanna region, the main part of northern Thailand, is a place of ethnic diversity. In this study, we investigated phak-gard-dong (PGD), or pickled mustard green (*Brassica juncea* L. Czern.), for its beneficial bacteria content and to analyse the variations in bacterial compositions among the PGD of three different ethnolinguistic groups, the Karen, Lawa, and Shan. DNA was extracted from the PGD pickled brine, and *16S rRNA* gene Illumina sequencing was performed. Metagenomic data were analysed and the results demonstrated that the dominant bacterial species were *Weissella* (54.2%, 65.0%, and 10.0%) and *Lactobacillus* (17.5%, 5.6%, and 79.1%) in the PGD of the Karen, Lawa, and Shan, respectively. *Pediococcus* was found only in the PGD of the Karen and Shan. Bacterial communities in PGD of the Lawa were distinctive from the other ethnic groups, both in the alpha and beta diversity, as well as the predicted functions of the bacterial communities. In addition, overall network analysis results were correlated to bacterial proportions in every ethnic PGD. We suggest that all ethnic PGDs have the potential to be a good source of beneficial bacteria, warranting its conservation and further development into health food products.

## 1. Introduction

Lanna, or the main part of northern Thailand, is an area in mainland South East Asia that has unique cultures; the area has also been settled by many ethnic minorities. The major ethnolinguistic group belongs to the Kra-Dai speaking family, which founded their state in the 13th century. Other groups are composed of Austroasiatic, Sino-Tibetan, and Hmong-Mien speaking families. Moreover, this area also receives cultural influence from two main civilizations of Asia, the Sinosphere and the Indosphere, which have had impacts on its cultures in various ways, including the food culture.

Foods and fermented foods of Lanna are unique from those of other parts of Thailand and the lowlands of South East Asia. The uniqueness is largely caused by its special mountainous landscape intersected by a number of major valleys and its inland tropical climates. With these, the Lanna inhabitants create their own cultures, which are mixtures of some major foreign influences, local cultures, and materials. Interestingly, in the midst of today’s modern world, in which manufactured food is widely consumed, the indigenous fermented foods of the Lanna region remain unique and popular in its local cultures. Some of the major indigenous fermented foods include fermented vegetables and fermented soybeans, which are part of the daily diets in all three ethnolinguistic groups in this area.

The technology to ferment vegetables is believed to have originated thousands of years ago in Asia and the Far East [1], especially in the Indian subcontinent, in the settlements that predate the great Indus Valley civilization [2]. The fermented products were made from various kinds of local vegetables [1]. This technology was introduced to Europe sometime in the 1500s, and fermented vegetables made of European cabbage varieties began to be produced [1]. Eventually, European settlers introduced cabbages and sauerkraut-making techniques to the New World [1]. Other fermented vegetables, particularly pickles and olives, have been produced and consumed in the Middle East, at least since biblical times [1]. Kimchi has become the most popular fermented vegetable in the Far East, particularly in Korea [1]. Presently, fermented vegetables are widely produced and consumed all around the world, with each region having its own unique fermentation process, creating products with distinctive flavours, although they are made from the same kind of vegetable. In Thailand, there are indigenous fermented vegetable products, such as Miang (fermented tea leaves from Miang Tea trees), Nam phak (fermented Chinese cabbage [*Brassica rapa* subsp. *chinensis*]), Tua nao (fermented soybean) [3], and phak-gard-dong (pickled mustard green). These products are associated with the Lanna people of Northern Thailand, despite the fact that the production processes and their appearances are similar to those of fermented vegetables from southern China and other parts of South East Asia [3].

Phak-gard-dong (PGD) is an ethnic fermented vegetable product of Thailand [4]. Various species of lactic acid bacteria, such as *Lactobacillus* sp., *Leuconostoc* sp., *Weisella* sp. and *Pediococcus* sp., are involved in the fermentation of PGD and other fermented vegetable products of many countries, for example, the kimchi of Korea [5,6,7,8], sinki and sunki of Japan [9,10,11,12], Pao cai and suan cai of China [13,14], and phak-sian-dong of Thailand [15,16].

Actually, fermented food studies in this area are not new topics, but the previous studies mostly focused on conventional methods of fermentation and utilisation of microbial isolates for product development [3,17,18,19]. The development of next-generation sequencing (NGS) has opened a new era of microbial community study to solve bias in the cultivation of culture-dependent methods and has also been adapted in the fermented food field. There is a large amount of research on well-known fermented foods. For example, the study of the biological, chemical, and physical properties of yogurts [20,21] and kimchi [22,23,24,25,26]. For Lanna fermented food, many research works in the microbial community focused on fermented tea (Miang) [27,28,29,30,31]. Another report by Pakwan and colleagues in 2020 surveyed four fermented foods of the Tai-Yuan ethnolinguistic group and their potential as functional foods via the NGS method [3]. However, comparative studies of microbial communities among ethnolinguistic groups are still lacking.

In this study, we compared bacterial communities in phak-gard-dong (PDG), one of the well-preserved fermented vegetable products of different Lanna ethnolinguistic groups. PGDs from three representative ethnolinguistic groups, the Karen (Sino-Tibetan), Shan (Kra-Dai), and Lawa (Austroasiatics), in the same geographical area, were studied using NGS technology. The data will reveal each ethnolinguistic group’s unique technology through modern scientific approaches and will contribute to a better understanding of ethnomicrobiology. Such an understanding could help promote local wisdom and assist in the conservation of ethnic fermentation technology and these beneficial indigenous fermented foods, which in turn will support food security in ethnic communities and contribute to food sustainability for humans worldwide. Moreover, the data may be used to improve the methods of fermentation and hygiene practices in food preparation to enhance food safety.

## 2. Materials and Methods

### 2.1. Sampling Sites and Sample Collection

#### 2.1.1. Sampling Sites

PGD was collected from 3 ethnolinguistic groups in Mae Hong Son, Thailand, in particular from (1) Karen at Ban Mueang Pam village, Pang Mapha district, (2) Lawa at Mae La Luang village, Mae La Noi district, and (3) Shan at Khun Yuam Saturday Market, Khun Yuam district. The samples were collected randomly from 5–6 households in Karen and Lawa villages and 6 shops in the Shan marketplace.

#### 2.1.2. PGD Fermentation Processes

**Figure 1:**
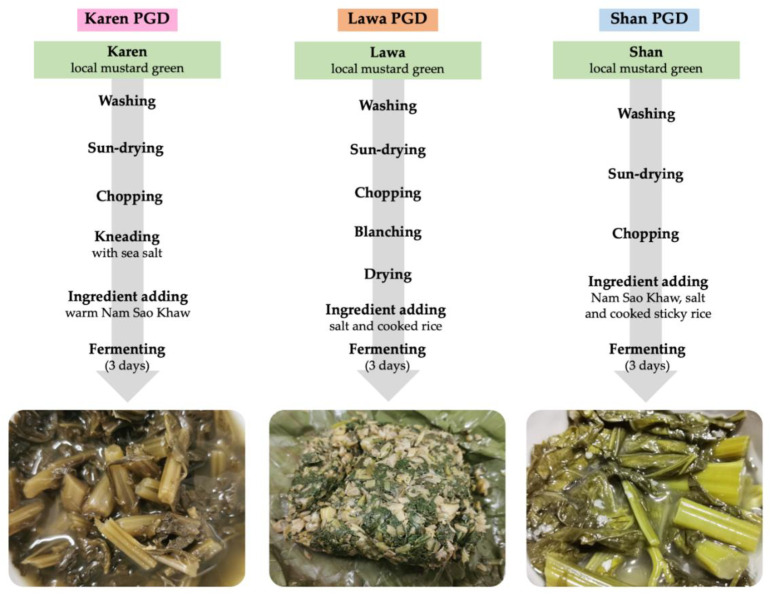
The PGD fermentation processes of the Karen, Shan, and Lawa, and characteristics of each ethnic PGD.

The PGD of Karen and Shan ethnic groups were processed as follows: first the mustard green was washed, then it was partly sun-dried until slightly wilting before being cut into small pieces and kneaded with salt. It was afterwards packed in a container with nam-sao-khaw (rinsed water from rice), and left to ferment for three days at room temperature (Figure 1). As for the PGD of the Lawa, after the mustard green was cut into small pieces, it was blanched in hot water and dried. Salt and cooked rice were then added and mixed, and the mustard green mixture was packed and left to ferment for three days (Figure 1).

#### 2.1.3. Sample Collection

Samples of PGD were collected after 3 days of fermentation. The samples from different households/shops were collected separately. The PGD samples were then collected in a centrifuge tube. DNA/RNA Shield (Zymo Research, Irvine, CA, USA) (10 mL) was added to the sample and the samples were kept at −20 °C until DNA extraction was performed.

#### 2.1.4. Determination of pH and Lactic Acid Bacteria Enumeration

A portion of each homogenised PGD sample was collected for pH measurement. Another portion of the sample (pickled brine, for the Karen and Shan PGDs; and the whole homogenate, for the Lawa PGD) was 10-fold diluted with 0.85% sodium chloride and spread on MRS agar, and supplemented with 0.05% L-cysteine and 0.004% bromocresol green (MRS-Cys-BCG agar). The agar was incubated at 37 °C for 48 h, and the lactic acid bacteria colonies, which are surrounded by a yellow zone, were counted and converted into log CFU/g or mL.

### 2.2. Total DNA Extraction

Each PGD sample in the DNA/RNA Shield was mixed using a vortex mixer. The liquid portion was centrifuged at 18,000× *g* for 30 min at 4 °C, then the liquid was removed and the pellet was collected for DNA extraction using ZymoBIOMICS™ DNA Miniprep Kit (Zymo Research, Irvine, CA, USA). DNA extraction from each sample collected from each household/shop was performed in duplicate and the extracted DNA was combined as one sample before sequencing.

### 2.3. DNA Sequencing

The extracted DNA for each sample that had a quantity of at least 2 ng/µL, purity (indicated by 260/280 absorbance ratio, A_260/280_) of ≥1.7 and volume of ≥30 µL was sent for next-generation sequencing of the *16S rRNA* amplicon (V3–V4 regions) using the Illumina platform with the Illumina MiSeq model. The sequencing library was constructed by randomly fragmenting the DNA sample and then ligating with Nextera XT Index primers, and sequencing was performed by Macrogen (Macrogen Inc., Seoul, Korea).

### 2.4. Sequence Processing and Analysis

The sequences of PGDs of the 3 ethnic groups were analysed with Qiime2 software [32]. Each sequence group was adapter trimmed (sequence of the V3-V4 region of the *16S rRNA* gene, including Bakt_341F: 5′-CCTACGGGNGGCWGCAG-3′ and Bakt_805R: 5′-GACTACHVGGGTATCTAATCC-3′). The trimmed sequences were quality-checked and denoised for amplicon sequence variants (ASVs) by DADA2 [33]. The ASV data was used to generate the rarefaction curve plot. The insertion tree was constructed, with replicate-sequence data and the green gene reference database version 13.8, to generate core metric results. The bacterial classification of ASVs was performed with the Greengenes classifier version 13.8, then, the taxa bar plots and taxa table of bacterial proportions were generated.

### 2.5. Alpha Diversity Analysis

The comparison of diversities of bacterial genera among the PGDs of the ethnic groups was performed using Qiime2 software with the command “qiime diversity core-metrics-phylogenetic” to generate core metric data, which included alpha diversity results.

### 2.6. Beta Diversity Analysis

Bacterial proportion data in the genus level was used in beta diversity analysis. The significance test of beta diversity was analysed using One-way PERMANOVA with the Bray-Curtis similarity index, and the non-metric multidimensional scaling (NMDS) plot was performed with PAST software version 4.03 [34].

### 2.7. Functional Analysis

Functional gene prediction of bacterial communities in PGDs was performed at the genus level by a phylogenetic investigation of the communities by reconstructing unobserved states 2 software (PICRUSt2) [35] based on the ENZYME nomenclature database [36]. The heatmap was visualised using R [37] and RStudio [37,38].

### 2.8. Network Analysis

Bacterial correlation was examined by R and RStudio [37,38] with “Hmisc” and “vegan” packages. The Fruchterman–Reingold plot was performed using Gephi 0.9.2 [39].

### 2.9. Sequences Deposit

Bacterial metagenomic sequences from the PGD were uploaded to the NCBI database under BioProject accession no. PRJNA747069.

## 3. Results

### 3.1. pH Measuring and Enumeration of Lactic Acid Bacteria

The pH of the PGDs of the Karen and Shan were 4.33 ± 0.03 and 4.63 ± 0.03, respectively. The pH of the Lawa PGD was significantly higher than the others, and was 5.95 ± 0.12. Likewise, the numbers of lactic acid bacteria found in Karen PGD (8.28 ± 0.03 log CFU/mL) and Shan PGD (8.24 ± 0.03 log CFU/mL) were similar, but in Lawa PGD, the number was lower (7.87 ± 0.03 log CFU/g) than that observed in the PGDs of the other groups.

### 3.2. Bacterial Community Analysis

Based on *16S rRNA* (V3-V4) sequences, the abundance of bacterial taxa in the PGDs was determined. In total, 1,706,873 sequences were detected across all PGD samples. After discarding low-quality sequences and denoising, a total of 1,068,999 sequences of 5987 ASVs were retained. ASVs were generated and taxonomic classification was performed using 97% identity reads and sequence depth at 52,500 as a minimum of frequency observed from the denoising.

### 3.3. Microbiome of Bacteria in Phak-Gard-Dong

**Figure 2:**
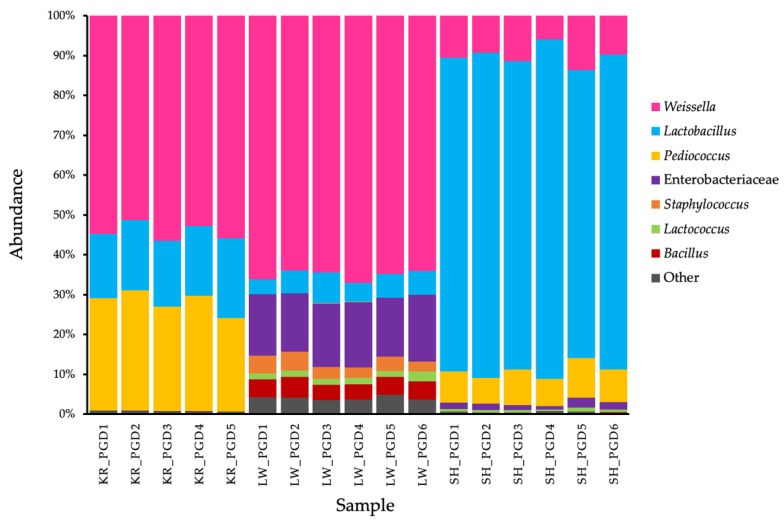
The proportion of bacterial taxa found in the PGDs of 3 ethnic groups including Karen (KR_PGD), Lawa (LW_PGD), and Shan (SH_PGD).

*Weissella* was the dominant bacterial population involved in lactic acid fermentation activity in PGDs, representing 54.2 ± 2.2% and 65.0 ± 1.2% of the total population in the PGDs of the Karen and Lawa ethnolinguistic groups, respectively, but only 10.0 ± 2.5% in Shan PGD. While *Lactobacillus* dominated the bacterial population in Shan PGD (79.1 ± 4.3%), it was only 17.5 ± 1.5% and 5.6 ± 1.3% in the PGDs of the Karen and the Lawa, respectively. Furthermore, *Pediococcus* was detected at a rate of 27.4 ± 2.6% and 8.0 ± 1.3% in Karen and Shan PGDs, respectively, but Lawa PGD contained less than 1% of this genus (Figure 2). Moreover, the bacterial community analysis at the species level demonstrated that *Weissella* was identified as *W. hellenica*, and *Lactobacillus* was identified as *L. paraplantarum*.

The bacterial communities of Karen and Shan PGDs were similar in terms of major bacterial taxa, including *Weissella*, *Lactobacillus*, and *Pediococcus*, but the proportions were different, whereas the proportions of *Weissella* and *Lactobacillus* were quite similar in Karen and Lawa PGDs.

### 3.4. Alpha and Beta Diversity

**Figure 3:**
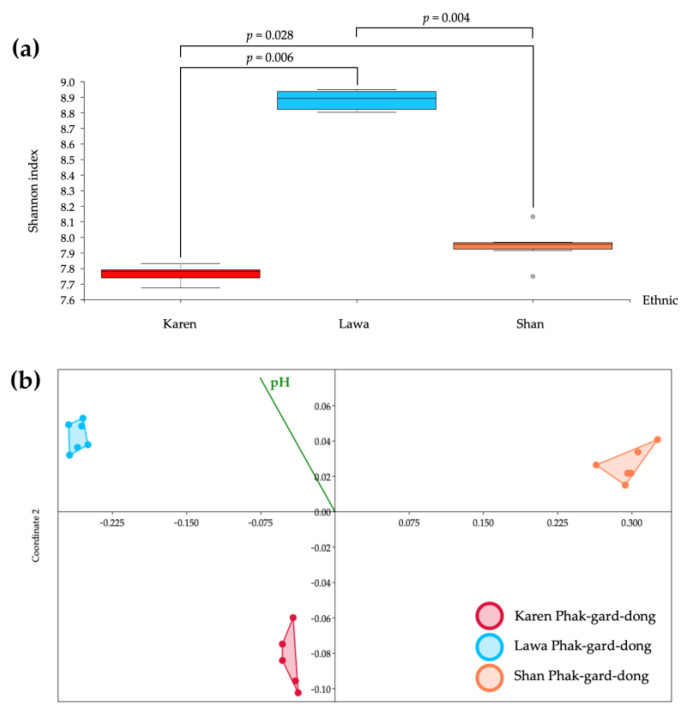
Alpha diversity boxplot with Shannon index demonstrated that the Karen PGD was different from Lawa PGD (*p* = 0.006) and Shan PGD (*p* = 0.028), and Lawa PGD was different from Shan PGD (*p* = 0.004) (**a**). The NMDS plot demonstrated the bacterial diversity differences among the PGDs from the three ethnolinguistic groups (**b**).

The alpha diversity, as measured by the Shannon index, indicated that Lawa PGD had the highest bacterial diversity, followed by Shan PGD and Karen PGD, respectively. Karen PGD had different bacterial communities than Lawa PGD (*p* = 0.006) and Shan PGD (*p* = 0.028), and Lawa PGD had different bacterial communities than Shan PGD (*p* = 0.004) (Figure 3a). The beta diversity analysis revealed the differences in the bacterial communities of Karen and Lawa PGDs (*p* = 0.0072), Karen and Shan PGDs (*p* = 0.0051), and Lawa and Shan PGDs (*p* = 0.006) (Figure 3b). These diversity analyses revealed significant differences in bacterial diversity and quantity among the PGDs of the ethnolinguistic groups, with Lawa PGD containing the most varieties, followed by Shan and Karen PGDs.

### 3.5. Functional Gene Prediction

**Figure 4:**
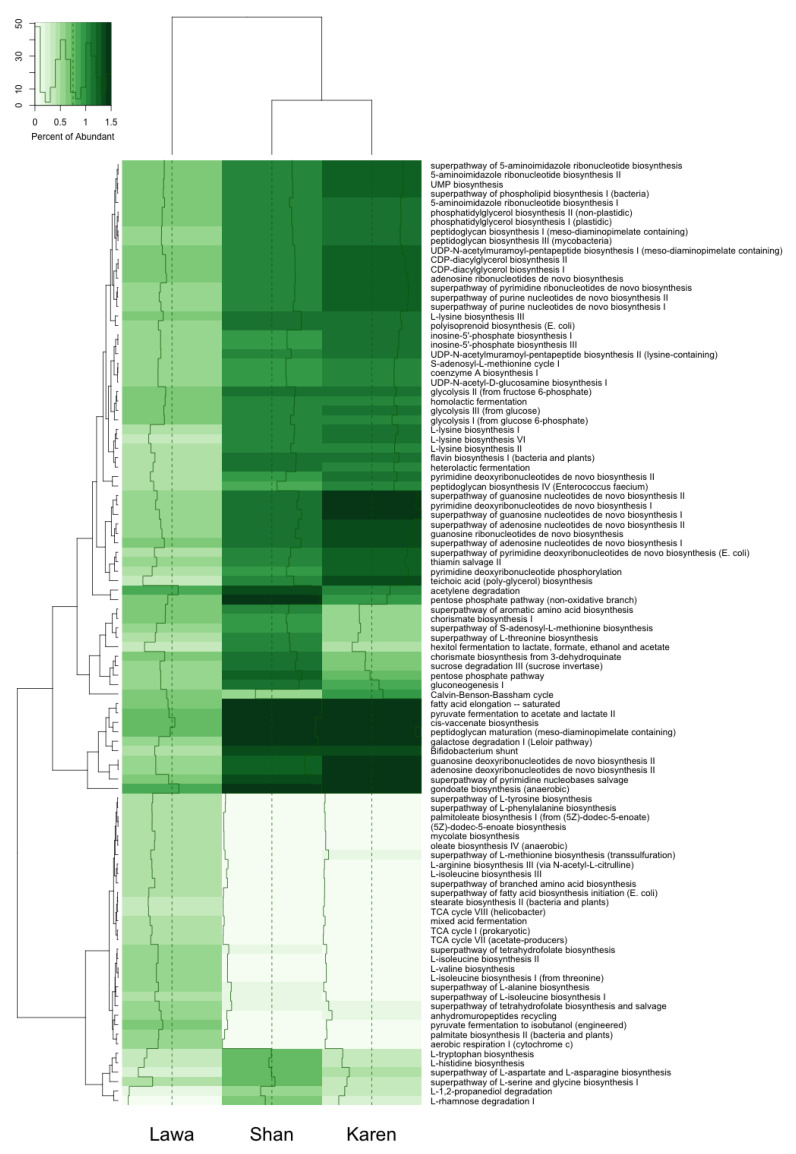
The functional genes heatmap of bacterial communities in the PGDs of the Karen, Lawa, and Shan. The predicted functional genes of the Shan and Karen PGDs and their abundance percentages were similar, while those of the Lawa PGD were different.

The top 100 functional genes predicted from bacterial communities in Karen and Shan PGDs demonstrated some similarities in the percentages of abundance, while the percentages of abundance of functional genes in the Lawa PGD were different from the others (Figure 4).

### 3.6. Network Analysis

**Figure 5:**
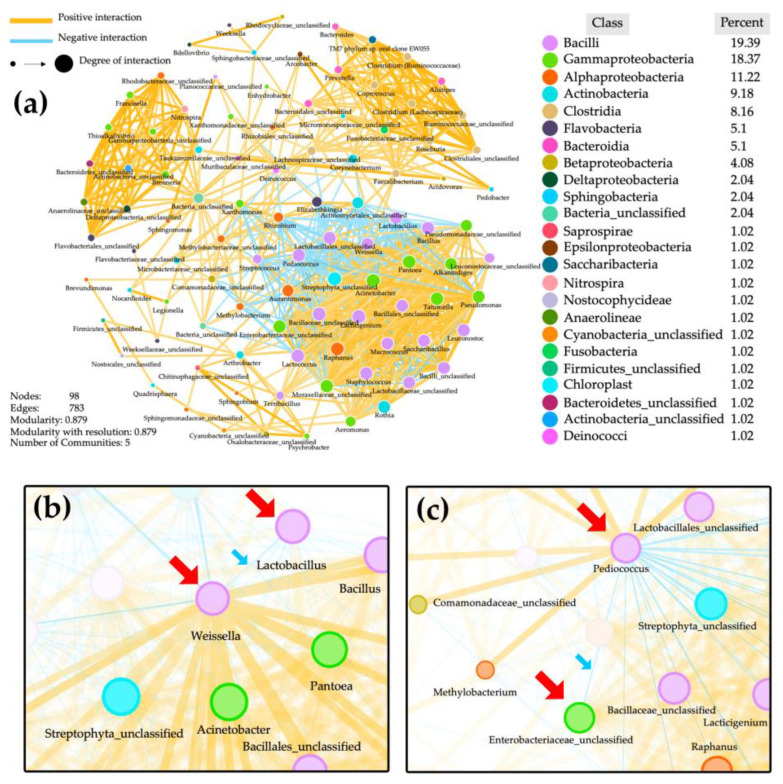
Network analysis of bacterial communities in PGDs, showing 98 nodes of bacterial identities with 783 edges of interactions between the bacterial taxa (**a**), and negative interactions (indicated by blue lines and blue arrows) between *Weissella* and *Lactobacillus* (**b**) and between *Pediococcus* and Enterobacteriaceae (**c**).

Network analysis showed the correlations between bacteria, with yellow and blue lines indicating positive and negative interactions between them (Figure 5). This plot demonstrated that among the five communities in PGDs, there were 98 nodes of 98 bacterial identities and 783 interactions among the bacteria within the communities. There were three major communities out of the five communities that were obviously observed, and the largest one contained the predominant bacteria. It demonstrated negative interactions between some predominant bacterial taxa, especially between *Weissella* and *Lactobacillus* (Figure 5b), and between *Pediococcus* and Enterobacteriaceae (Figure 5c).

## 4. Discussion

PGD is made by fermenting mustard green leaves with naturally occurring endophytic and exophytic bacteria found in/on the leaves. Sea salt and nam-sao-khaw (rinsed water from rice) or rice was added in the fermentation process, which are the sources of sodium and carbohydrates, respectively. They provide nutrients for bacterial growth. Salt can also inhibit the growth of clostridia [40,41] and other aerobic bacteria [42]. PGDs are usually fermented for at least 3 days prior to consumption.

Natural bacteria adhered to mustard green leaves play important roles in the fermentation process. Certain bacteria can grow in the presence of the additional ingredients. The PGD flavours are derived from acidic bacterial metabolites, such as lactic acid produced during fermentation by lactic acid bacteria. Lactic acid bacteria, especially *Weissella* and *Lactobacillus*, were found to be the most prevalent genera in the three ethnic PGDs. Kamdee et al. (2014) and Jeong et al. (2013) found that *Weissella* was the predominant bacterium during early fermentation, but was eventually replaced by *Lactobacillus* during later stages of fermentation [43,44]. In our study, the PGDs used in this experiment had been fermented for 3 days. *Weissella* was the predominant bacterium in Karen PGD (54.2 ± 2.2%) and Lawa PGD (65.0 ± 1.2%), but in Shan PGD, *Lactobacillus* was the predominant genus (79.1 ± 4.3%). There are various factors that influence the growth of different genera of lactic acid bacteria, including the additional ingredients used in various recipes, the fermentation process and fermentation temperature in various locations, and the type and numbers of bacteria naturally found on mustard green leaves in various areas. A previous study reported that *Weissella* was also discovered in a variety of Chinese traditional pickled vegetables, including peppers, eggplants, garlic, cucumbers, radish, mustard root, and assorted vegetables [45]. This genus has been reported to co-exist with *Lactobacillus* in many fermented foods [44,46,47].

In addition to *Weissella* and *Lactobacillus*, which are the predominant species in the three ethnic PGDs, *Pediococcus* was also found as a predominant genus in the Karen and Shan PGDs. Moreover, we found small proportions of enterobacteriaceae and *Lactococcus* in Shan PGD, and Enterobacteriaceae, *Lactococcus*, *Staphylococcus*, and *Bacillus* in Lawa PGD. These differences in predominant bacterial taxa in each ethnic PGD may be the result of the differences in indigenous bacteria in each local area and in the fermentation processes. The alpha diversity showed the most bacterial varieties in Lawa PGD, followed by Shan PGD and Karen PGD, respectively, which were significantly different. There were also significant differences in bacterial varieties among the three ethnic PGDs, as supported by the NMDS plot and one-way PERMANOVA.

There are many similar fermented vegetable products to PGD, which use similar raw materials or processes, in many countries. Suan cai or pickled Chinese cabbage began to be fermented by *L. oligofermentans*, *L. curvatus*, *L. mesenteroides*, and *Acinetobacter* sp. around day 6 of fermentation. Then, on day 30 of fermentation, *L. curvatus*, *L. plantarum*, and *L. oligofermentans* were the predominant bacteria in Suan cai [48]. Additionally, *L. acidophilus*, *L. fermentum*, *L. casei*, and *L. lactis* were found in Suan cai [48,49,50]. Kimchi contains a variety of beneficial bacteria, including *Lactobacillus*, *Leuconostoc*, and *Weissella* as the predominant bacterial genera [8,47]. In fermented turnip leaves, or sunki in Japan, *Lactobacillus*, *Pediococcus*, and *Leuconostoc* were found as predominant bacteria [51,52]. In our study, the predominant bacteria found in PGDs were *Weisella*, *Lactobacillus*, and *Pediococcus*, which are similar to the bacteria found in the fermentation of Korean kimchi (*Weissella* and *Lactobacillus*) and Japan’s sanki (*Lactobacillus* and *Pediococcus*). Karen, Lawa, and Shan PGDs, however, are more similar to kimchi in terms of the proportions of *Weissella* and *Lactobacillus*, as shown in the study of Park et al. (2012) [53]. This suggests that the indigenous bacteria found in various vegetable species in different countries may be similar, and although there are different fermentation processes of fermented vegetable products in each region, the unique fermentation products could have similar predominant microorganisms.

Functional prediction demonstrated that the potential bacterial community’s activities in Lawa PGD were different from those in Karen and Shan PGDs. Heterolactic fermentation and glucose fermentation, which produces lactic acid, carbon dioxide, and ethanol [54], and pyruvate fermentation, which produces acetate and lactate, were found in Karen and Shan PGD bacterial activities more than in the Lawa PGD. In contrast, mixed acid fermentation activity in Lawa PGD was shown with higher percentages of abundance than in the Karen and Shan PGDs. The mixed acid fermentation is related to enterobacteriaceae and occurs when bacteria utilise two or more different pathways during the fermentation’s final stages [55,56]. The bacterial activities predicted for different PGDs of the ethnolinguistic groups reflected the types and proportions of the bacterial populations, which could be contributed by factors such as temperature, fermentation process, and fermentation ingredients, as mentioned above.

In the bacterial network analysis (Figure 5), when focusing on the predominant bacterial genera found in PGDs, *Weissella* and *Lactobacillus* were negatively correlated (Figure 5b). This could explain why the amounts of *Weissella* and *Lactobacillus* are inverse; i.e., in the PGDs with high amounts of *Weissella*, *Lactobacillus* was detected in low amounts, as seen in the Karen and Lawa PGDs. By contrast, *Weissella* was detected in low amounts in the PGD of Shan, whereas *Lactobacillus* was detected in higher relative abundances (Figure 2). Likewise, the negative relationship was found between *Pediococcus* and enterobacteriaceae (Figure 5c). A significant amount of enterobacteriaceae contamination was observed in Lawa PGD, correlating to a very small amount of *Pediococcus*. While *Pediococcus* was more abundant in the Shan and Karen PGDs than in Lawa PGD, enterobacteriaceae was less abundant or absent (Figure 2). Additionally, *Pediococcus* was unrelated to *Weissella* and *Lactobacillus*, and thus had no effect on them. Although *Weissella* and *Lactobacillus* seemed to have an antagonistic effect on one another, both genera are lactic acid bacteria that could have a major role in PGD fermentation.

Due to the fact that the PGDs in this study were produced by three ethnolinguistic peoples using traditional methods, fermented vegetable production might be contaminated with harmful bacteria. Because the production process can take place in a low hygiene environment, it is easy for the PGDs to be contaminated by pathogenic bacteria from other sources, including untreated water used for watering vegetables, improper washing, and improper handling steps, which might have been the reasons for significant proportions of enterobacteriaceae and *Staphylococcus*, especially in Lawa PGD. Manufacturers should consider the cleanliness of raw materials, equipment, and premises to avoid contamination by pathogenic bacteria.

PGDs have the potential to be health foods due to their beneficial bacteria content. According to previous studies, several species of *Weissella* have probiotic properties, including tolerance to low pH and to bile salts resembling the gastric pH and bile concentration in the small intestine [57,58,59]. Additionally, our study revealed that *Wiessella* found in PGD was *W. hellenica*, a species that was reported to have the ability to produce many bacteriocins [59,60,61,62,63]. Production of bacteriocins is one of the probiotic properties and bacteriocins can potentially be natural food preservatives. Moreover, *Lactobacillus* found in this study was identified with Greengenes database as *Latobacillus paraplantarum*. This bacteria was also found in Korean pickled “Jangajji” [64] and many strains were examined and found to exhibit probiotic potential properties, including stability under intense gastric and bile conditions, and exhibit antioxidant and immunostimulatory activities, anti-inflammatory activities, and antimicrobial activities [64,65,66,67,68,69]. The presence of these bacteria in PGDs in our study indicated not only the presence of bacteria with probiotic potential, but also the presence of bacteria capable of producing bacteriocins, which can extend the shelf life of food.

Considering the bacterial community patterns, the proportions of predominant bacteria, especially *Weissella* and *Lactobacillus*, in Karen PGD were similar to those in Lawa PGD, whereas proportions of these bacteria were obviously different in Shan PGD. Moreover, in the research work of Punchay and colleagues in 2020, Karen’s wild food plants were found to be similar to Lawa’s wild food plants [70], although the number of species was different. Therefore, whether the mustard green was spread from one ethnolinguistic group to another or whether it was indigenous to each region, it is likely that the mustard green’s endemic bacteria are similar. When mustard green was fermented, the predominant bacteria occurred with approximately the same proportions and differed slightly in the various locations. However, there are no reports about the relationship between the wild food plants of Shan and Karen or of Shan and Lawa. However, Lawa PGD was unique and distinct from the bacterial communities of others. The proportions of enterbacteriaceae, *Staphylococcus*, and *Bacillus* were significantly higher than the others (Figure 2). Moreover, their Shannon diversity index was also far higher than the other two groups (Figure 3a), and the ordination plots of the three PGDs were distinctive from one another (Figure 3b). Considering their fermentation processes in Figure 1, the Karen and Shan PGDs were processed differently from the Lawa PGD. After cutting mustard greens into small pieces, Shan and Karen recipes continue the process by mixing directly with nam-sao-khaw and salt, then letting it ferment in a mixed solid–liquid phase. The Lawa mustard green was blanched with hot water and then dried before the addition of salt and cooked rice, and finally fermented in a semi-dry condition in a banana leaf wrap. In general, as low oxygen conditions are more favourable to lactic acid bacteria, the container of the Lawa PGD, which may have had a higher oxygen concentration, might have been one of the contributing factors to the lower proportions of lactic acid bacteria and higher proportions of other bacteria, in addition to possible contamination during the process. Our results of lactic acid bacteria enumeration on MRS agar also revealed a significantly lower number of lactic acid bacteria (Table S1). The greater diversity of bacteria in the Lawa PGD and the lower proportions of lactic acid bacteria were also correlated to its higher pH values (Table S1). We suggest that this unique process of Lawa PGD allowed non-lactic acid bacteria, especially the enterobacteriaceae, *Staphylococcus* and *Bacillus*, to grow in their PGD during the fermentation process with the lactic acid bacteria and made its bacterial community structure different from those in the PGDs of the other two ethnic groups.

This study revealed that these ethnic PGDs are also good sources of beneficial bacteria and demonstrates their potential to be super foods in the future. We suggest conserving this kind of food for consumption in the local area, but to improve the hygiene process. This local and traditional wisdom and knowledge is one way to solve the problem of nutrition poverty and support food sustainability for ethnic societies.

## 5. Conclusions

In this study, we found that the PGDs of the Karen (representative of Sino-Tibetan) and Shan (representative of Kra-Dai) were similar to each other, while the PGD of the Lawa (representative of Austroasiatics) was distinctive from the other PGDs in alpha and beta diversity, as well as the predicted functions of the bacterial communities. The greater diversity of bacteria in the Lawa PGD and the lower proportions of lactic acid bacteria were also correlated to its higher pH values. The bacterial genera that were predominant in the PGDs of the three groups were *Weissella* and *Lactobacillus*. In addition, *Pediococcus* was found in the PGDs of the Karen and Shan. The PGDs from all three ethnolinguistic groups, on the other hand, have the potential to be health foods, containing potential probiotic bacteria. This suggests that the PGDs of these ethnolinguistic groups should be conserved and should be further developed into health food products for wider consumption.

## Data Availability

Publicly available datasets were analysed in this study: This data can be found under BioProject accession number: PRJNA747069.

## Supplementary Materials

The following supporting information can be downloaded at https://www.mdpi.com/article/10.3390/biology11010150/s1, Table S1. The pH values and amount of lactic acid bacteria in Karen, Lawa, and Shan phak-Gard-Dong (PGD) at day 3 of fermentation.
