# Supplementary material for: Bacterial Communities in Lanna Phak-Gard-Dong (Pickled Mustard Green) from Three Different Ethnolinguistic Groups in Northern Thailand
Source: Biology (Basel). 2022 Jan 17;11(1):150. doi: 10.3390/biology11010150 (PMC8772952; doi:10.3390/biology11010150)
Supplement: Supplementary file 1 [file biology-11-00150-s001.zip › biology-1509743-supplementary.pdf]

Table S1. The pH values and amount of lactic acid bacteria (LAB) in Karen, Lawa, and Shan Phak-gard-dong (PGD) at day 3 of fermentation.

Sample	pH	LAB (log CFU/g or mL)
Karen PGD	4.33±0.03	8.28±0.03
Lawa PGD	5.95±0.12	7.87±0.03
Shan PGD	4.63±0.03	8.24±0.03
